# Knowledge, attitude and self-efficacy program intended to improve public health professionals’ ability to identify and manage perinatal depressive symptoms: a quasi-experimental study

**DOI:** 10.1186/s12889-020-10086-9

**Published:** 2020-12-30

**Authors:** Nitikorn Phoosuwan, Pranee C. Lundberg

**Affiliations:** 1 Department of Public Health and Caring Sciences, Faculty of Medicine, Uppsala University, BMC, Husargatan 3, Box 564, 751 22 Uppsala, Sweden; 2 Department of Community Health, Faculty of Public Health, Kasetsart University Chalermphrakiat Sakonnakhon Province Campus, Chiang Khruea sub-district, Muang Sakon Nakhon, Sakon Nakhon Province Thailand

**Keywords:** Attitude, Healthcare professionals, Knowledge, Public health professionals, Self-efficacy, Thailand, Training program

## Abstract

**Background:**

During the perinatal period women lack screening and treatments for perinatal depressive symptoms, while public health professionals (PHPs) in primary care centres (PCCs) need training for identification and management of such symptoms. This quasi-experimental study was aimed at evaluating knowledge, attitudes and self-efficacy among PHPs after participating in a Knowledge, Attitude, and Self-efficacy (KAS) program for identification and management of perinatal depressive symptoms.

**Method:**

The KAS-program, carried through in Sakonnakhon in north-eastern Thailand, comprised one day of theory and a four-week period of field practice. Thirty-three PHPs from PCCs participated in the program. Twenty-three of them participated in focus group discussions (FGDs). Chi-square for trend, paired-sample T-tests and content analysis were used.

**Results:**

Knowledge, attitude and self-efficacy scores increased after the PHPs had fully participated in the KAS-program. Four categories emerged from the FGDs: increased understanding and knowledge, being aware and having a positive attitude, having confidence and ability to work, and need of regular training and feedback.

**Conclusion:**

The KAS-program may contribute to giving PHPs in PCCs the knowledge, positive attitude and self-efficacy they need to identify and manage perinatal depressive symptoms. Implementation of the KAS-program to other healthcare professionals such as nurses/midwives is great of interest.

**Supplementary Information:**

The online version contains supplementary material available at 10.1186/s12889-020-10086-9.

## Background

Depression is one of the most common mental disorders occurring in women during the perinatal period [1, 2]. Worldwide, nearly 40% of women experience psychological distress due to depressive symptoms during the perinatal period [3]. If women with perinatal depressive symptoms are under-screened and untreated they may suffer from negative short-term as well as long-term effects [4], such as reduced mother-to-child attachment and reduced self-efficacy [5].

There are a large number of empirically-supported interventions for treating perinatal depression in at-risk women in primary care and obstetrics/gynecology practices. There are also interventions such as counseling intervention, psychosocial or psychological intervention and interpersonal psychotherapy from other clinical settings [6–8]. Training is important also for mental health practitioners. Training for them, aimed at improving their knowledge and attitudes, may be a strong factor for better recovery [9, 10]. As improved knowledge and attitudes may not last over time, their training may need to take place steadily or repeatedly [10–13]. Nonetheless, there is lack of screening, and often women with depressive symptoms have not been well-managed during the perinatal period [4, 8]. Improving women’s mental health is a part of the third of the sustainable development goals, viz. reduction of premature mortality [14], and women need improved identification and management of depressive symptoms during the perinatal period [7, 8, 15].

Healthcare professionals (HCPs) are responsible for management of depressive symptoms in primary care centers (PCCs) and antenatal care (ANC) clinics [15, 16]. However, they do not always accurately identify depressive symptoms in pregnant women [4]. In part, this is due to their lack of familiarity with standardized depression screening tools, such as the Edinburgh Postnatal Depression Scale (EPDS) [17]. In addition, HCPs may lack knowledge about the process of referral and diagnostic assessment [3, 10].

The EPDS is a recommended screening tool that is applicable in PCCs [18]. Public health professionals (PHPs) in PCCs are important persons for effective provision of mental health care [19, 20]. Therefore, improving the ability of PHPs in PCCs to identify perinatal depressive symptoms among women by use of the EPDS is important [17].

In Thailand, depression screening has been launched in PCCs, and women in the perinatal period are classified in a high-risk group for depression [21]. Each of the 76 provinces in Thailand has several PCCs, and every PCC is responsible for a population of 3000-8000. About four HCPs work in each PCC; two PHPs work with disease prevention and health promotion, and one nurse/midwife and one public health assistant work with common illnesses and rehabilitation of patients [22]. PHPs should work toward identification and management of perinatal depressive symptoms among women during the perinatal period [21]. However, the PHPs need capacity-building programs devoted to knowledge of and attitude towards management of perinatal depressive symptoms among women who receive ANC in their PCCs [21, 23, 24]. As tools for screening such symptoms are essential for PHPs, a program focusing on identification of perinatal depressive symptoms could improve their self-efficacy [25].

Research has demonstrated a link between attitudes and behavior, with attitudes viewed as antecedents of behavior [26]. In order to change a behavior among HCPs, understanding of their attitudes toward that behavior and training are necessary [27]. Currently, very little research has been focused on attitudes and behavior among HCPs, such as those who work with identification and management of perinatal depressive symptoms at primary care setting [3, 26].

The knowledge of HCPs is related to their attitude, which in turn is related to their self-efficacy. The self-efficacy of HCPs is a key factor for improving women’s mental health because women may trust and follow guidance from those who have high level of self-efficacy [24, 28]. The knowledge of HCPs may be low if they have insufficient experience of a specific task or lack of training [9, 23]. Their attitude can be improved through a training program [9, 25, 29].

According to the theory of self-efficacy [30], this concept focuses on efficacy expectations, comprising four sources of information: performance accomplishment, vicarious experience, verbal persuasion, and physiological states. Knowledge and attitude may contribute to performance accomplishment; vicarious experience comes when a person succeeds in a specific task, supervision enhances verbal persuasion, and physiological states come from a non-verbal action from an expert. If HCPs in PCCs have sufficient knowledge, positive attitude and high self-efficacy to identify and manage perinatal depressive symptoms, women may have better chance to access healthcare services and to be treated [4, 18]. A training program targeting PHPs in PCCs may be of great interest for improvement of PHPs’ knowledge, attitude and self-efficacy in order to make them identify and manage perinatal depressive symptoms effectively [10, 17, 19]. To improve knowledge, attitude and self-efficacy among PHPs for identification and management of perinatal depressive symptoms, a knowledge, attitude and self-efficacy program (KAS-program) was developed as a training program for this study.

## Methods

### Aim

The aim of this study was to evaluate knowledge, attitude and self-efficacy among PHPs in PCCs in north-eastern Thailand after they had participated in a KAS-program for identification and management of perinatal depressive symptoms.

### Study setting and design

Data were collected in Sakonnakhon, a northeastern province of Thailand. Sakonnakhon has about 300 PHPs, with a bachelor’s degree in public health, working at 168 PCCs. The number of childbirths annually is high [31]. This quasi-experimental study utilized mixed methods approach [32, 33] in which quantitative method was used to demonstrate the changes of knowledge, attitude and self-efficacy scores of PHPs for identification and management of perinatal depressive symptoms during the KAS-program, while qualitative method provided a rich source of information to understand the process during and after implementation of the KAS-program.

### Knowledge, attitude, and self-efficacy (KAS) program for identification and management of perinatal depressive symptoms

**Fig. 1:**
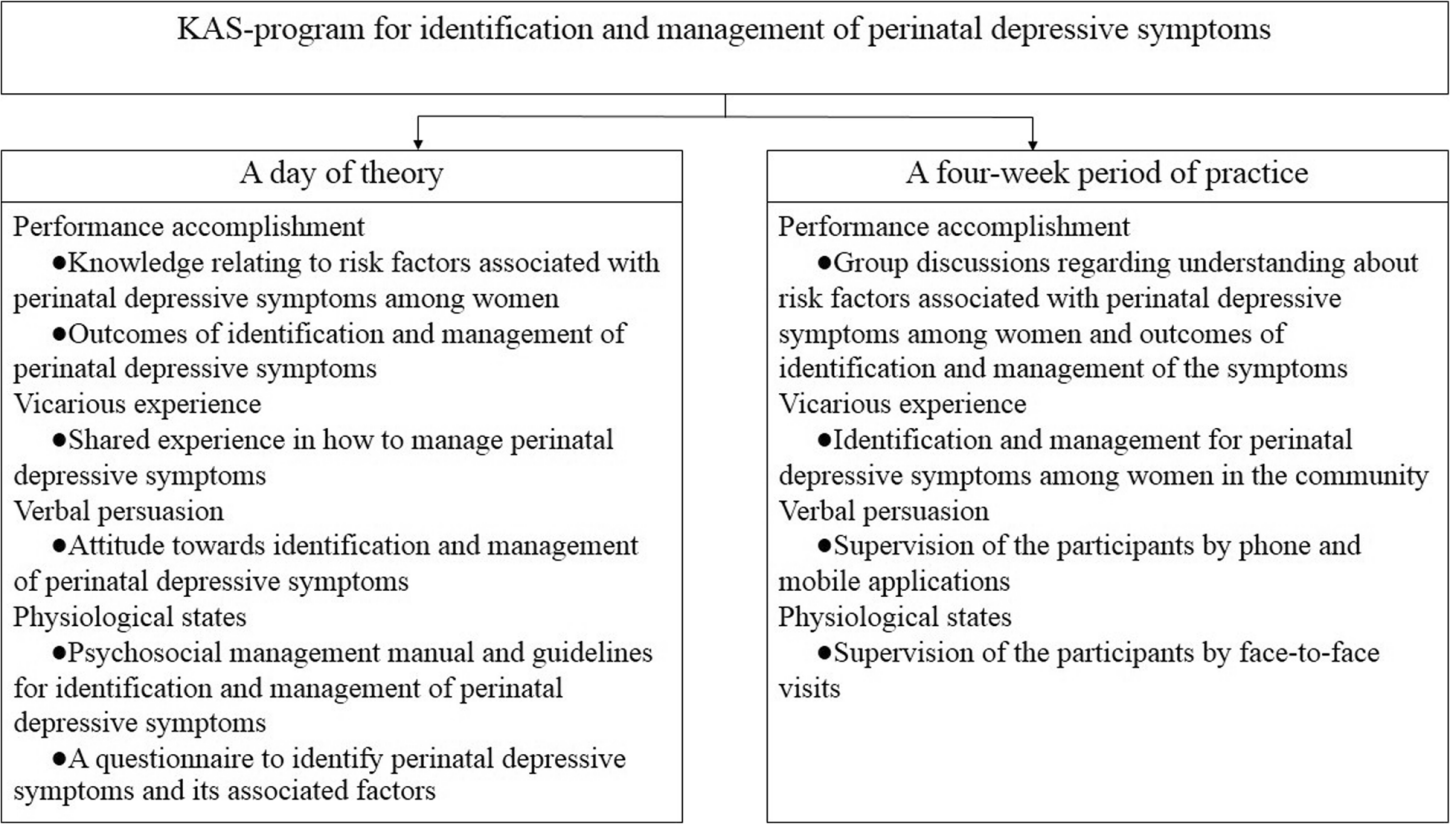
Contents of the Knowledge, Attitude and Self-Efficacy Program (KAS-program) in relation to the four main components of the Bandura’s self-efficacy theory [30]

The KAS-program was developed by the authors (NP and PCL) on the basis of the theory of self-efficacy [30]. It contained two parts: (1) a full day (8 h) of theory and (2) a four-week period of field practice. See Fig. 1.

The day of theory included interactive lectures provided by three experts in the fields of public health, behavioral science and psychiatric nursing. It focused on knowledge of, attitude towards and self-efficacy for identification and management of perinatal depressive symptoms. Two guidelines were used. One from the World Health Organization [34], translated into Thai language by NP, dealt with psychosocial management. Another, constructed by the authors, was devoted to identification and management of perinatal depressive symptoms. A questionnaire used among north-eastern Thai women, was also included. It comprised four parts: (1) the Thai EPDS part for screening women during the perinatal period [1, 5, 35, 36], (2) the psychological well-being question part [37], (3) the self-esteem question part [38] and (4) the sense of coherence question part [1, 39]. The participants were given the guidelines and a copy of the questionnaire for practice.

The four-week period of field practice started immediately after the day of theory. During 4 weeks, the participants were asked to practice with two women or more (one pregnant woman and one woman after childbirth) visiting the ANC clinic at their PCC. For this practice they used the guidelines and the questionnaire presented in the day of theory. In order to assist the participants, NP supervised them by use of mobile applications (Line and Facebook). Each participant was also contacted once by telephone. Face-to-face visits were arranged by NP for those who requested.

### Participants

The participants were PHPs employed at PCCs in six selected districts of Sakonnakhon (*N* = 18) with large numbers of childbirths in Sakonnakhon in recent years [31]. There was no specific criteria for selecting the participants in this study. We calculated the sample size [40]. Mean differences and standard deviations (SDs) (based on our pilot study in a province near Sakonnakhon) were: knowledge score = 1.2 and 1.12, attitude score = 4.0 and 3.11, and self-efficacy score = 4.0 and 4.3; β was 80% and α was 0.05. After some losses, the final number of participants was 33.

After the four-week period of field practice, we invited by telephone all participants from the KAS-program to take part voluntarily in four focus group discussions (FGDs). Twenty-three PHPs agreed to participate in focus groups as follows: FGD1 (*n* = 8), FGD2 (*n* = 6), FGD3 (*n* = 5), and FGD4 (*n* = 4). Ten participants could not participate in the FGDs because of, e.g., urgent tasks when the FGDs were held.

### Procedure

We submitted the proposed research study to the Ethics Committee for approval, while the heads of the Health Promotion Department of Sakhonnakhon Provincial Public Health Office and of the District Public Health Offices approved the KAS-program and the data collection. Thereafter we sent, by ordinary post, invitation letters together with socio-demographic characteristics questionnaires (concerning age, gender, marital status, level of education, training experience in mental health field, size of workplace, and working experience in PCCs) and documents about the study to PHPs in PCCs in the six selected districts of Sakonnakhon. One hundred and thirty-four PHPs agreed to participate in the KAS-program and signed a consent form. Later, PHPs who had agreed were selected to participate in the KAS-program using a simple randomization method. The selected PHPs were informed about their participation in the day of theory at the Sakhonnakhon Provincial Public Health Office.

**Fig. 2:**
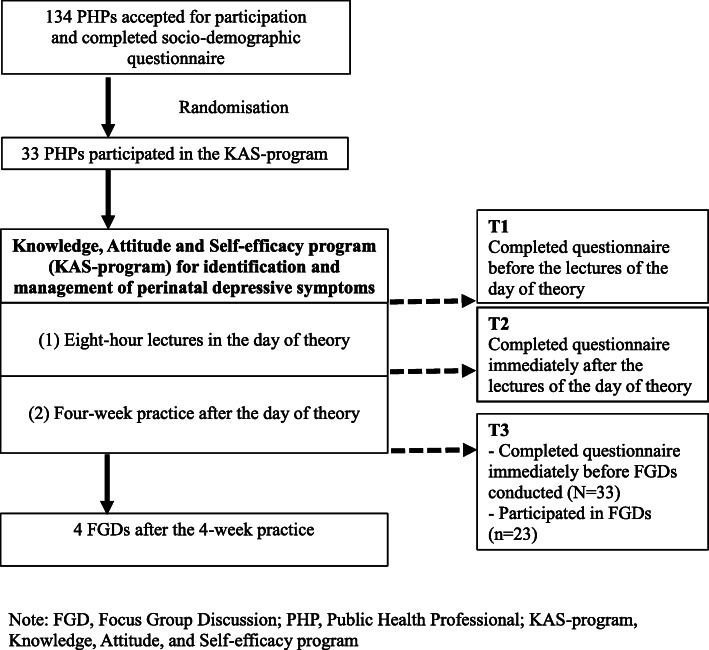
Flow diagram for the quasi-experimental study

On the day of theory, the authors informed the participants about the aims of the study, their rights as participants in the KAS-program and their option to withdraw from the KAS-program. The participants were asked to three times complete a questionnaire about knowledge, attitude, and self-efficacy for identification and management of perinatal depressive symptoms: (T1) before the first lecture in the day of theory, (T2) after the last lecture in the day of theory, and (T3) immediately after finishing the field practice before the FGDs or on a day close to the last FGD (in any FGD in cases of non-participation). See Fig. 2.

The FGDs were conducted in district public health offices in Sakonnakhon at a time and date the participants preferred. Each FGD, with NP and PCL present, was audio-recorded and lasted approximately 2 h. After this time, no new information emerged. NP, male author with a degree in public health and experience of qualitative research, served as moderator. PCL, female author, nurse/midwife and PhD with experience of qualitative research, took notes and clarified answers from the participants when needed. The authors had no previous relation to the participants. After each FGD, NP transcribed the recordings verbatim and checked the accuracy of the transcripts. All transcripts were coded without name identification.

### Instruments

A questionnaire was developed for this study by the authors (see Additional file 1). It consisted of three parts related to perinatal depressive symptom identification and management: (1) knowledge of, (2) attitude towards, and (3) self-efficacy for such activities in their profession. Three experts approved the content of the questionnaire (Content Validity Index: CVI > 0.80). Internal consistency of the questionnaire was tested with 30 PHPs who worked in PCCs nearby Sakonnakhon (the Cronbach’s alpha coefficient for the whole questionnaire was 0.96).

The knowledge and attitude parts were based on a questionnaire intended to train Thai PHPs and public health assistants [41]. Adjustments for the purpose of this study were made by the authors. Twelve questions concerned knowledge of perinatal depressive symptom identification and management. Each question could give a score of zero or one if the answer was incorrect or correct, respectively. Thus, the total score of this part could be 0–12, and a higher score meant more knowledge. Ten questions concerned attitude towards identification and management of perinatal depressive symptoms. Each question had a four-level Likert-scale: Strongly agree, agree, disagree, and strongly disagree, and could be scored between one and four. Thus, the total score could be 10–40, and a higher score meant more positive attitude.

The self-efficacy part was based on Thai validated version [42] of the Generalized Self-Efficacy Scale [43]. Adjustments for the purpose of this study were made by the authors. It had ten items, each with four options (not at all true, hardly true, moderately true, and exactly true), providing a score 1–4. Thus, the total score could be 10–40, and a higher score meant higher self-efficacy.

An interview guide for use in the FGDs was developed for this study by the authors. The interview guide contained eight open-ended questions letting the participants in the FGDs describe and discuss their knowledge of, attitude towards, and self-efficacy for identification and management of perinatal depressive symptoms after participation in the KAS-program. It was tested in the first FGD and adjusted before use in the remaining FGDs. The first FGD was involved in the qualitative analysis. Example questions were: (1) “How has your knowledge changed after you participated in the KAS-program?” and (2) “Please share your experiences from participating in the four-week period of field practice of the KAS-program”.

### Analyses

Different statistics were used for analyses of the questionnaire. Descriptive statistics were applied to summarize socio-demographic characteristics of the participants using frequency, percent, mean, SD, and range. Assumptions for paired-sample T test were satisfied (i.e., continuous variables, independent observations, normal distribution shown by skewness values was between − 0.7 and 0.5, and no outliners defined by kurtosis values were less than 2). Therefore, Chi-square test for trend analysis was used to determine changes of mean scores of the knowledge, attitude and self-efficacy over three time points, and paired-sample T test was used to compare the mean scores between times. The significance level was set at 0.05, and 95% confidence interval was used.

Data from FGDs were analyzed using qualitative content analysis [44]. It focused on meaning units (words or sentences), condensed meaning units, codes, sub-categories and categories, where the categories represented the processes during and after implementation of the KAS-program. First, the authors read all transcripts separately. Thereafter, they discussed categories until agreement was reached. The categories were reported to the participants, and the feedback from them did not result in any adjustments. Example quotations were translated from Thai to English and back by the authors who were Thai natives with good knowledge of English. They are shown in the result section with indication of the focus group (FGD1–4).

## Results

Most participants were female (66.7%) and single (57.6%). Two-thirds of them were at most 30 years old, their age ranging from 22 to 54 years. See Table 1.

**Table 1:** Characteristics of participants in the study

Characteristics of participants	N (percent)
Gender	
Female	22 (66.7)
Male	11 (33.3)
Age (years)	
≤ 30	20 (60.6)
31–40	8 (24.2)
> 40	5 (15.2)
Mean (SD) = 32.2 (7.60), Range = 22–54	
Marital status	
Single	19 (57.6)
Married	13 (39.4)
Widowed	1 (3.0)
Training experience in mental health field	
No	30 (90.9)
Yes	3 (9.1)
Working duration in a PCC	
1–5 years	15 (45.4)
6–10 years	12 (36.4)
> 10 years	6 (18.2)
Mean (SD) = 7.1 (5.31), Range = 1–26	
Size of current PCC	
Small	4 (12.1)
Medium	22 (66.7)
Large	7 (21.2)

Note: *PCC* Primary care centre; *SD* Standard Deviation

### Quantitative results

The knowledge scores of perinatal depressive symptom identification and management increased significantly from 8.94 at T1 (before lectures the day of theory) to 9.45 at T2 (after lectures the day of theory), and remained the same from T2 to T3 (after 4 weeks of field practice) (p for trend = 0.031). The attitude scores toward identification and management of perinatal depressive symptoms improved significantly from 28.94 at T1 to 29.88 at T2, and from T2 to 30.42 at T3 (p for trend = 0.004). Self-efficacy scores for perinatal depressive symptom identification and management were unchanged from 26.79 at T1 to 26.33 T2, and showed improvement from T2 to 28.73 at T3 (*p* = 0.043 for trend). See Table 2.

**Table 2:** The differences of scores of knowledge of, attitude towards and self-efficacy for perinatal depressive symptom prevention between times

Items	Mean ± SD				Changed T1 to T2		Changed T2 to T3	
	T1	T2	T3	p for trend†	mean (95%CI)	*p*-value	mean (95%CI)	*p*-value
Knowledge	8.94 ± 0.75	9.45 ± 0.75	9.42 ± 1.12	.031*	0.52 (0.22, 0.81)	.001*	0.03 (−0.41, 0.35)	.872
Attitude	28.94 ± 3.17	29.88 ± 4.21	30.42 ± 2.54	.004*	0.94 (0.09–1.78)	.031*	1.52 (0.39–2.64)	0.010*
Self-efficacy	26.79 ± 3.87	26.33 ± 4.12	28.73 ± 2.95	.043*	−0.45 (−1.45, 0.55)	.363	2.39 (1.23, 3.56)	<.001*

Note: *SD* Standard Deviation; T1, before lectures in the theoretical day; T2, after lectures in the theoretical day; T3, before the FGDs conducted; *CI* Confidence interval; †, obtained by Chi-square trend analysis

* statistically significant at 0.05 level

### Qualitative results

Four categories related to PHPs having participated in the KAS-program emerged: increased understanding and knowledge, being aware and having a positive attitude, having confidence and ability to work, and need of regular training and feedback.

#### Increased understanding and knowledge

All FGD participants described that after the KAS-program they had increased their understanding of perinatal depressive symptoms among women. Most participants without previous knowledge of perinatal depression indicated that after participating in the theoretical and practical parts of KAS-program they had gained knowledge. Also, a few participants with mental health education indicated increased knowledge. The participants mentioned that the program was valuable because it made them pay more attention to the mental health of women during pregnancy and after childbirth. It was important to identify pregnant women for antenatal depressive symptoms and manage postpartum depressive symptoms by giving them help and support. The participants described that pregnant women coming to ANC clinics used to have a pink book of mother-and-child health called “Samud Anamai Manda”, and they received a mental health screening called 2Q with only two questions at their first ANC visit. Usually, no mental health problem or depression was found among pregnant women by use of 2Q. If a mental health problem of a pregnant woman was found, she was referred to hospital. The participants also found that compared with 2Q, the screening tool from the KAS-program was very useful for evaluation of depressive symptoms of pregnant and postpartum women.

After participating in this program, I have increased my knowledge of mental health during pregnancy and after childbirth. … I did not have this knowledge before. I can use it in my work. (FGD1).

I know more about screening of perinatal depressive symptoms, particularly in pregnant women. Before my work was about screening for depression among teenagers, from 15 years, without focus on pregnant women. I think I have got a new method that is helpful for screening work after the program. (FGD2).

The program is good for us (PHPs). We have increased our understanding of mental health among women. I have received a manual for prevention of perinatal depressive symptoms to use. I know that women can have depressive symptoms from pregnancy to 1 year after childbirth. (FGD4).

#### Being aware and having a positive attitude

All participants described that after the KAS-program they had increased their awareness and changed their attitude towards perinatal depression. They mentioned that they had a more positive attitude than before to work with this problem. They described that when pregnant women come to ANC clinics they focused on the women’s physical health and carried out pregnancy examinations. Therefore, they could not see if pregnant women had depressive symptoms. When their attitude had been changed they considered working with the women’s mental health by talking, listening and giving advice.

After the program, I have opened my mind to have a new perspective on women’s depressive symptoms during pregnancy and after delivery. I have a positive attitude to work that supports the women. (FGD1).

I think we realize this problem (perinatal depressive symptoms) after the program. Before, I believed that the symptom could disappear by itself after delivery. As PHPs we should consider and detect this problem and promote mental health. (FGD2).

#### Having confidence and ability to work

All participants indicated that they had more self-confidence after participating in the KAS-program. They believed that they could work with identifying depressive symptoms by using the manual they had received from the program. They mentioned that their work was disease prevention. Therefore, they should cooperate with nurses/midwifes at PCCs and in this way work with perinatal depression as a team and close to people in their community, particularly women during pregnancy and after childbirth. They described that according to the Ministry of Public Health in Thailand, the important work of primary care should be “located close to where people live” (Klaibarn, Klaijai). Therefore, they worked together with nurses/midwives at PCCs to approach women closely concerning mental health promotion in their community.

Our work is prevention of diseases in the community. We know everybody and they trust us. When we visit villages in the community, we could screen depressive symptoms among pregnant women to prevent these symptoms after delivery. (FGD1).

I have more confidence after participating in the program. I believe that I could work with prevention of perinatal depressive symptoms. I will work together with nurses/midwifes to screen women during pregnancy and after delivery. (FGD3).

#### Need of regular training and feedback

In the KAS-program, the participants received knowledge for identification and management of perinatal depressive symptoms based on theory and also on practice through screening and its evaluation. They mentioned that it had been very useful to receive coaching during the fieldwork of their practice. However, they would have appreciated getting more such coaching, discussion and feedback. Regular training for identification and management of perinatal depressive symptoms should be provided to PHPs to update their knowledge and increase their self-confidence. The participants also mentioned that nurses/midwives should participate in this training program.

I think it will help us if we would have more time for coaching while we practice screening in the fieldwork. (FGD2).

I think we are aware of perinatal depressive symptoms and have more knowledge than before but I think PHPs and nurses/midwives still need regular training in this area so that we can work with women’s mental health promotion. (FGD4).

## Discussion

This study revealed that the PHPs had generally no or little knowledge about identifying and managing perinatal depressive symptoms before participating in the KAS-program. This is in accord with several studies showing that the knowledge of Thai HCPs for a specific task is often insufficient [23, 24], particularly the knowledge for management of depressive symptoms in communities [21]. In this study, knowledge scores for identification and management of perinatal depressive symptoms among PHPs increased immediately after the participants had finished their participation in the interactive lectures in the day of theory. Thereafter, the scores remained on the same level till after the 4 weeks of field practice. Further, at the end of the KAS-program the PHPs described their understanding of how to identify and manage perinatal depressive symptoms and to use the EPDS for women. Perhaps the guidelines and the questionnaire presented in the lectures of the day of theory filled a knowledge gap of the PHPs, while field practice during 4 weeks maintained the PHPs’ knowledge for identification and management of perinatal depressive symptoms. A specific training program can increase the knowledge of HCPs [9], while a knowledge gap of HCPs for identification of depression is filled if HCPs are advocated for depression screening in women [17]. Having enough knowledge can also make HCPs’ able to improve mental health services in a PCC, particularly HCPs may improve psychosocial management among women during the perinatal period [6, 16, 19]. Nowadays, public primary healthcare services in Thailand have the same structure and organization, e.g., ANC clinics [22]. The KAS-program is of interest for dissemination also to other HCPs, like nurses/midwives [6]. Therefore, HCPs in PCCs can have sufficient knowledge to identify and manage perinatal depressive symptoms in the future [21, 23].

When HCPs perform screening routinely for perinatal depressive symptoms and feel a screening tool is useful, they may have a positive attitude to work with identification of such symptoms [17]. The KAS-program demonstrated gradual changes of the attitude scores toward identifying and managing perinatal depressive symptoms, from before lectures in the day of theory longitudinally till after 4 weeks of practice. The participants also described having more positive attitude and awareness to work with perinatal depressive symptoms. Their attitude might be changed by (1) shared experience and lectures provided in the day of theory based on the four components of the Bandura’s self-efficacy theory [30], and (2) their supervised practice during 4 weeks. It is necessary for HCPs to manage perinatal depressive symptoms [6]. However, they need a training program within 5 years, otherwise their attitudes may not improve [9]. The attitude among HCPs in Thailand is often negative when they take care of specific patients [24, 25]. The KAS-program seemed to improve the PHPs’ attitude towards identification and management of perinatal depressive symptoms and made them recognize the importance of this task. A training program focusing on attitudes among HCPs, like the KAS-program, should be managed and implemented on a policy level [26].

Our results demonstrated improvement not only of the participants’ self-efficacy scores for identification and management of perinatal depressive symptoms, but also of their ability to work with perinatal depressive symptoms after finishing the KAS-program. Four main components of Bandura’s self-efficacy theory (i.e., performance accomplishment, vicarious experience, verbal persuasion, and physiological states) contribute to self-efficacy and they should be included in training programs [30]. Performance accomplishment and vicarious experience are important components of increasing HCPs’ self-efficacy [28], while verbal persuasion and physiological states are key points that make HCPs have self-efficacy to provide mental health services [19]. In this study, the four main components were considered and used both in the day of theory and during the 4 weeks of field practice. The participants described that they had confidence and ability to identify and manage perinatal depressive symptoms because during 4 weeks, they practiced with two or more women during the perinatal period. They also expressed that they would get more self-confidence if they had a regular training and the opportunity of talking with other HCPs, e.g., nurses/midwifes. Moreover, there was an increasing trend of the participants’ self-efficacy scores from before the lecture of the day of theory till after 4 weeks of practice. Increased practice in PCCs can promote high self-efficacy among HCPs, while regular training may enhance the ability to perform mental health tasks [28]. In Thailand, a follow-up session can improve outcomes of a training program [29], and regular training along with supervision is a challenge in PCCs in Thailand in order to enhance competency of HCPs to manage perinatal depressive symptoms [23, 25]. Therefore, the KAS-program in this study is important for PHPs at primary care settings to improve their knowledge, attitudes and self-efficacy. This program should be useful also for other HCPs such as nurses/midwives at primary care settings and at hospitals. Future research should evaluate the KAS-program using smartphone applications that were found to be useful in treatment of perinatal depression [45, 46].

### Strengths and limitations

The study relied on Bandura’s self-efficacy theory. Effectiveness of a training program could be well demonstrated by using a quasi-experiment with mixed methods data collection approach [47]. Sample calculation and data analysis were carried out in order to evaluate quantitatively the knowledge, attitude and self-efficacy for identification and management of perinatal depressive symptoms. Although a number of studies have shown that training can improve the capacity among nurses and physicians to identify and manage perinatal depressive symptoms, we have not been able to find similar studies for PHPs in PCCs. Therefore, the contribution of this study to existing knowledge is unique.

A limitation was that the KAS-program was followed up on a four-week basis. Increased time for follow-up might show a greater change of knowledge, attitude and self-efficacy. For further study of the effectiveness of the program there is need of a control group.

## Conclusions

The KAS-program, based on four main components of Bandura’s self-efficacy theory, had both a day of theory and a four-week period of practice. The program increased the knowledge, attitude, and self-efficacy scores among PHPs in PCCs for identification and management of perinatal depressive symptoms. The participating PHPs had increased knowledge and awareness, positive attitude, and increased confidence and ability to work. However, they wished to have regular training and feedback after finishing the KAS-program. It is recommended to implement the KAS-program nationally also to other HCPs such as nurses/midwives.

## Supplementary Information

**Additional file 1.**

## Data Availability

The datasets used and/or analysed during the current study are available from the corresponding author on reasonable request.

## Abbreviations

ANC: Antenatal care
CVI: Content validity index
EPDS: Edinburgh postnatal depression scale
FGD: Focus group discussion
HCP: Healthcare professional
KAS-program: Knowledge, attitude and self-efficacy program
PCC: Primary care centre
PHP: Public health professional
SD: Standard deviation
T1: Before the first lecture in the day of theory
T2: After the last lecture in the day of theory
T3: Immediately after finishing the field practice
