# Supplementary material for: Knowledge, attitude and self-efficacy program intended to improve public health professionals’ ability to identify and manage perinatal depressive symptoms: a quasi-experimental study
Source: BMC Public Health. 2020 Dec 30;20:1926. doi: 10.1186/s12889-020-10086-9 (PMC7774237; doi:10.1186/s12889-020-10086-9)
Supplement: Supplementary file 1 — Additional file 1. [file 12889_2020_10086_MOESM1_ESM.pdf]

Knowledge related to perinatal depressive symptom identification and management

Please use √ for true statements and × for false statements.

.... 1. Depression is ranked top 10 among Thai females of 'Years of Life Lost due to Disability' (YLD).

.... 2. Prevalence of antenatal depressive symptoms among women, in Thailand and in other countries, is about 5%.

.... 3. Pregnant women have a reduced risk of developing depressive symptoms compared to non-pregnant women because women's emotions change to be more positive during pregnancy.

.... 4. Depressive symptoms can occur during pregnancy and thereafter until one year after childbirth.

.... 5. Depressive symptoms among pregnant women include physical, emotional, cognitive and behavioral symptoms.

.... 6. Pregnant women who are depressed can have reduced appetite and increased wish to sleep.

.... 7. Genetics is the main cause of depressive symptoms among pregnant women.

.... 8. Risk factors associated with antenatal and postnatal depressive symptoms among women are low psychological well-being, low self-esteem, low sense of coherence and teenage pregnancy.

.... 9. According to the principles of depressive symptoms screening, the questions should not be explained or expanded. If the participants don't understand, the questions can be repeated.

.... 10. Cognitive and behavioral adjustment is a way of preventing antenatal depressive symptoms among women.

.... 11. The best way of caring for pregnant women at risk for depressive symptoms is to use antidepressant drugs.

.... 12. A consequence of depressive symptoms among pregnant women is reduced child development.

Attitudes related to perinatal depressive symptom identification and management

Please check √ in the box related to your attitude

Statements	Strongly agree	Agree	Disagree	Strongly disagree
1. I am sympathetic to pregnant women who are depressed.				
2. Because of their behavior I don't give services to pregnant women with depressive symptoms.				
3. I often feel that depressive symptoms depend on the moods of the depressed pregnant women.				
4. I am often moody when giving services to pregnant women with depressive symptoms as they too often think negatively.				
5. Depressive symptoms among pregnant women are unimportant because these symptoms will disappear after childbirth.				
6. Explaining to depressed pregnant women that they have physical disorders, such as having low or high blood pressure or having a risk of diabetes, is more convenient than explaining that they have a mental problem.				
7. Prevention of depressive symptoms among pregnant women is a duty of the physicians, nurses or psychologists.				
8. Caring of pregnant women with depressive symptoms take longer time than caring of women with physical symptoms. Referring women with depressive symptoms to a specialist should be emphasized.				
9. Antidepressant drugs are an easy way of preventing and treating depressive symptoms.				
10. Capacity building among health personnel in the area of antenatal depressive symptoms among women may increase the quality of services for pregnant women.				

Self-efficacy related to perinatal depressive symptom identification and management

Please check √ in the box related to your opinion in relation to perinatal depressive symptom identification and management

Statements	Not at all true	Hardly true	Moderately true	Exactly true
1. I can always manage to solve difficult problems if I try hard enough.				
2. If someone opposes me, I can find the means and ways to get I want.				
3. It is easy for me to stick to my aims and accomplish my goals.				
4. I am confident that I could deal efficiently with unexpected events.				
5. Thanks to my resourcefulness, I know how to handle unforeseen situations.				
6. I can solve most problems if I invest the necessary effort.				
7. I can remain calm when facing difficulties because I can rely on my coping abilities.				
8. When I am confronted with a problem, I can usually find several solutions.				
9. If I am in trouble, I can usually think of a solution.				
10. I can usually handle whatever comes my way.				
